# 3D Bioprinting as a Powerful Technique for Recreating the Tumor Microenvironment

**DOI:** 10.3390/gels9060482

**Published:** 2023-06-12

**Authors:** Ilaria Parodi, Donatella Di Lisa, Laura Pastorino, Silvia Scaglione, Marco Massimo Fato

**Affiliations:** 1 Department of Computer Science, Bioengineering, Robotics and Systems Engineering, University of Genoa, 16126 Genoa, Italy; donatella.dilisa@edu.unige.it (D.D.L.); laura.pastorino@unige.it (L.P.); marco.fato@unige.it (M.M.F.); 2 National Research Council of Italy, Institute of Electronic, Computer and Telecommunications Engineering (IEIIT), 16149 Genoa, Italy; silvia.scaglione@ieiit.cnr.it; 3 React4life S.p.A., 16152 Genova, Italy

**Keywords:** bioprinting, tumor microenvironment, hydrogels

## Abstract

In vitro three-dimensional models aim to reduce and replace animal testing and establish new tools for oncology research and the development and testing of new anticancer therapies. Among the various techniques to produce more complex and realistic cancer models is bioprinting, which allows the realization of spatially controlled hydrogel-based scaffolds, easily incorporating different types of cells in order to recreate the crosstalk between cancer and stromal components. Bioprinting exhibits other advantages, such as the production of large constructs, the repeatability and high resolution of the process, as well as the possibility of vascularization of the models through different approaches. Moreover, bioprinting allows the incorporation of multiple biomaterials and the creation of gradient structures to mimic the heterogeneity of the tumor microenvironment. The aim of this review is to report the main strategies and biomaterials used in cancer bioprinting. Moreover, the review discusses several bioprinted models of the most diffused and/or malignant tumors, highlighting the importance of this technique in establishing reliable biomimetic tissues aimed at improving disease biology understanding and high-throughput drug screening.

## 1. Introduction

Cancer is the second leading cause of death worldwide, immediately after heart disease [1]. Despite the progress achieved over the years in early diagnosis, a deep understanding of the biological mechanisms behind cancer behaviors remains an open challenge, as well as the discovery of new anticancer therapies. Numerous evidence suggests that the mechanisms within the tumor microenvironment (TME) are deeply involved in drug resistance [2]. One of the main reasons is the fact that the tumor microenvironment is a very heterogeneous and dynamic system that includes cancer cells, the extracellular matrix (ECM), blood vessels, immune cells, fibroblasts, macrophages, and many other cells. Every TME internal modification is involved in tumor progression.

**Figure 1:**
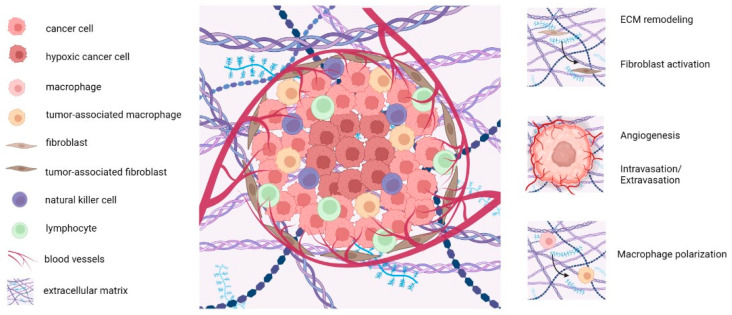
Schematic representation of the tumor microenvironment (TME): legend with the cellular and extracellular components of the TME (**left**); TME structure (**center**); some biological phenomena occurring within the TME (**right**). Created with BioRender.com.

Cancer-associated fibroblasts (CAFs) promote tumorigenesis and ECM remodeling by stimulating the secretion of various ECM proteins, such as hyaluronic acid and collagen. Moreover, immune cells, tumor-associated macrophages (TAMs) and natural killer cells (NKs) play essential roles in cancer growth through several inflammatory responses (Figure 1). Compared with healthy tissues, the tumor stroma exhibits a stiffer extracellular matrix, containing growth factor and signaling molecules that are continually secreted [3]. Besides the stiffness of the matrix, some other mechanical cues, such as solid stress, may influence the diffusion of drugs that is usually heterogeneous within the tumor mass [4]. In fact, solid stress could result in the compression and deformation of blood and lymphatic vessels, the enhancement of fluid pressure, and ECM remodeling. In this regard, both its stiffening and degradation due to matrix metalloproteinases (MMPs) can have a great impact on tumor progression, thus also promoting metastatic processes [5,6].

Moreover, the TME exhibits an oxygen gradient, leading to the formation of a hypoxic core where cancer cells have been shown to be able to survive in these harsh conditions, switching their metabolism, consuming more glucose, and acidifying the environment [7]. Furthermore, most traditional chemotherapeutic agents target only the normoxic cells, leading to a strong impact in terms of drug resistance and treatment inefficiency. In fact, oxygenated cells that are close to the vasculature are killed by the treatment flowing within the vasculature tree, while hypoxic cells typically confined in the central area of the tumor may survive and regenerate the tumor [8]. The hypoxic condition, in turn, leads to the formation of new blood vessels, which are often dilated, convoluted, excessively branched and leaky because the high proliferation of tumor cells is faster than the angiogenesis. This condition causes, as for the solid stress, an increase in interstitial fluid pressure in the tumor core [9].

To enhance the biological mechanisms behind cancer progression, 2D cell cultures have been widely used because of their simplicity and low cost; however, they have a limited capacity in recapitulating the in vivo tumor microenvironments due to the absence of the spatiotemporal complexity previously described, neglecting the interactions between the cancer cells and the surrounding extracellular matrix [10]. Cancer research studies have also been carried out using animal models which, despite a better resembling of the tumor solid features with respect to bi-dimensional cell cultures, exhibit biological differences with humans, resulting in poor clinical outcomes [11]: for these reasons, according to a recent statistical study, only 3.4% of anticancer drugs pass all clinical phases [12].

Hence, different in vitro three-dimensional (3D) cancer models using biomimetic extracellular matrices (e.g., hydrogels) have been produced to study cancer biology and reach more reliable clinical results in drug screening [13]. Among the various techniques to produce complex and more realistic 3D cancer models is bioprinting, which allows the realization of spatially controlled hydrogel-based scaffolds, easily incorporating different types of cells.

The purpose of this review is to discuss the various 3D bioprinting techniques and biomaterials employed. Moreover, different tumor tissue models will be presented, highlighting the importance of 3D bioprinting as an innovative and powerful tool in cancer research.

## 2. Bioprinting Methods

**Figure 2:**
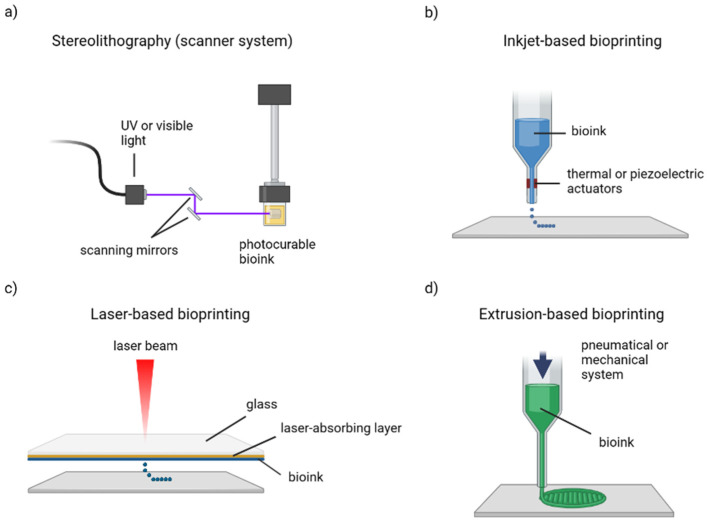
Schematic representation of the most common bioprinting methods. (**a**) Stereolithography with scanner mirrors that crosslink each layer point-by-point. (**b**) Inkjet-based bioprinting produces droplets through thermal or piezoelectric actuators. (**c**) Laser-based bioprinting employs a laser beam to create droplets starting from a bioink ribbon. (**d**) Extrusion-based bioprinting realizes continuous bioink filaments. Created with BioRender.com.

A proper three-dimensional model capable of recapitulating the overall characteristics of cancer tissue is, in fact, crucial for a deep understanding of tumor behavior and in the development of new anticancer therapies [13]. Today, bioprinting represents an important emerging technology in tissue engineering not only for oncological research due to the possibility of creating complex models with high spatial control, recreating a biomimetic cancer microarchitecture [14], but also for regenerative medicine, cosmetics testing and even, over the last years, in the food industry [15,16,17]. In general, bioprinting consists of the deposition of a biomaterial in a predetermined spatial manner, adopting the same approach as a classic 3D printer. During the last years, different approaches have been adopted in the field of bioprinting; however, the most popular methodologies include stereolithography, inkjet-based bioprinting, laser-assisted and extrusion-based bioprinting (Figure 2) [18]. In this section, these methodologies are presented, with some applications in cancer modeling, and their main properties are then summarized in Table 1.

### 2.1. Stereolithography

Stereolithography is based on the crosslinking of a photosensitive material, using ultraviolet rays as a light source, ensuring, thanks to their high energy, an extremely rapid process, which can take place in two different ways. In the laser-based method, the curing of each layer is obtained point-by-point through a scanner system; meanwhile, the digital light projection relies on a digital micromirror device to project and then crosslink an entire cross-section of the three-dimensional structure [19]. Stereolithography has been diffused in tissue engineering for its high speed, especially using the digital light projection method, and for its high resolution of up to 25 µm. Moreover, being a nozzle-free technique, no shear stress is exerted on cells, and thus cell viability is maintained high (>85%) after the printing process [20]. Since the cytotoxicity of some photoinitiators represents a limiting factor of cell viability, some scientists have already begun to investigate photoinitiator-free biomaterials or visible light-absorbing photoinitiators.

This technique does not require particularly stringent rheological properties, and a wider range of viscosities can be used compared to other bioprinting types. In general, the use of low viscosity material (0.25 and 10 Pa·s) and low cell density is useful for limiting the light scattering and facilitating the removal of the unreacted, thus not cured, bioink [21]. However, the biomaterial should be sufficiently viscous to avoid cell settling during crosslinking. This bioprinting strategy offers the opportunity to explore multimaterial integrations with fewer concerns over the undesirable voids between material domains.

Moreover, stereolithography also provides access to architectures, such as hollow vessels and branching structures, which would otherwise be difficult to realize with traditional biofabrication methods. In this context, Miri et al. [22] incorporated a microfluidic device into a stereolithographic bioprinter to switch between different bioinks and produced different complex biological constructs, including a tumor angiogenesis model, composed of a GelMA matrix laden with breast cancer cells and tortuous channels filled with vascular endothelial cells (ECs). Grigoryan et al. [23] also developed a multimaterial stereolithographic bioprinter that uses an automated biomaterial selection process with the aim of creating heterogeneous structures, keeping a manual rinsing step in order to prevent unwanted mixing. Such technology was used as a first step to investigate murine lung adenocarcinoma in a core–shell structure, showing a controllable matrix invasion, and secondly, to study the cooperation between two sub-populations in heterogeneous mosaic architectures. Despite an enhanced printing time due to the manual rinsing process, this bioprinter allows the creation of larger constructs.

### 2.2. Inkjet-Based Bioprinting

Inkjet-based bioprinting dispenses bioink in a drop-on-demand manner onto a substrate, mainly using thermal or acoustic forces. In thermal inkjet bioprinting, bioink is heated, and the vapor generated forces it out of the nozzle. The heating temperature is usually 200–300 °C; however, the overall bioink temperature rises to a maximum of 10 °C above room temperature due to the very short heating time [24]. According to several studies, cell viability is about 70–95%, relying on the cell phenotype and printing parameters. The typical droplet diameter is in the range of 30–80 µm. In the case of acoustic forces-based bioprinters, a piezoelectric actuator produces sound waves that break the bioink, leading to the drop extrusion. During piezoelectric bioprinting, the droplets are larger than those generated by the thermal method, ranging from 50 to 100 µm. The creation of the droplets by the acoustic method must take place in a frequency range not exceeding 15–25 kHz to prevent damage to the cell membrane [25]. However, with the right printing parameters, the survival rate of cells is higher than 90% [26]. Overall, inkjet bioprinting can produce up to 10,000 droplets per second, which is a highly desirable quantity in high-throughput screening applications, such as drug testing and cancer screening [27].

Some typical drawbacks of inkjet bioprinting are the mandatory use of low cell densities and low viscosity bioinks, typically lower than 10^6^ cells/mL and about 3.5–12 mPa·s, respectively, in order to avoid nozzle clogging [24,28]. Further disadvantages of this technique include the nonuniformity of droplet size and the bioprinting of multiple cell phenotypes at the same time. To overcome this last limitation, Zhang et al. [29] have successfully developed a custom-made inkjet, printing with four channels, enabling the simultaneous ejection of four different types of cells. The integration with a microfluidic device was employed to evaluate a co-culture of glioblastoma and liver carcinoma cells and to conduct drug metabolism and diffusive studies.

### 2.3. Laser-Assisted Bioprinting

Laser-assisted bioprinting is a nozzle-free, noncontact method where a laser beam is pulsed on a three-layer ribbon: laser-transparent glass, laser-absorbing metal, such as titanium or gold, and bioink. The laser beam energy is absorbed by the ribbon, which rapidly generates a local bubble on the opposite side which, in turn, ejects a desired amount of bioink on a receiving substrate [30]. This technique is applicable for high cell densities (10^7^–10^8^ cells/mL), and this is important, for example, when producing a vascular system with ECs. The resolution of the laser-assisted bioprinting can reach 10–100 µm and relies on several factors, such as the thickness of the absorbing layer and the bioink layer coated onto the ribbon, the viscosity and surface tension of the bioink, wettability of the substrate, laser energy, as well as the air gap between the ribbon and the receiving stage. The viscosity range of the bioink is in the range of 1–8000 mPa·s [31], while lasers with wavelengths from 193 to 1064 nm are employed. If the laser energy is too low or the bioink viscosity is too high, the jet cannot be developed completely, which may result in no material transfer; therefore, these two parameters are fundamental for the success of the printing. Being a nozzle-free approach, laser-assisted bioprinting does not impart mechanical shear stress on cells, which exhibit a high viability (>95%) after the printing process [32]. Bioprinters with a high-frequency pulse laser can obtain high-throughput printing up to 5 kHz, thus 5000 droplets per second.

The drawbacks of this method are related to the high cost of the printer and to the absorbing layer: the break due to the intense energy absorbed can generate fragments that can contaminate the bioink and damage the living cells. This can be avoided by using a short pulse duration and low laser energy or employing a double-absorbing layer, introduced by Lin et al. [33], consisting of a sacrifice-adhesive layer and a metallic foil one. The researchers used this method to directly print human colon cancer cells, demonstrating a better printing resolution compared with other laser-based technologies, allowing the formation of smaller cell-laden droplets and higher cell viability due to the lower contamination from the laser energy-absorbing material. In addition to this, the preparation of the ribbon is a time-consuming step, especially when using different biomaterials or cell phenotypes [24].

### 2.4. Extrusion-Based Bioprinting

Extrusion-based bioprinting represents the most popular method for the biofabrication of tissues. Although droplets and microbeads can be produced, bioink usually is extruded in the form of a continuous cylindrical filament through a nozzle or a needle and deposited layer-by-layer onto a print bed. The extrusion bioprinters exploit either pneumatic or mechanical dispensing systems [34]. In some bioprinters, the print head can move in all three spatial dimensions; in others, it can move in the x- and y-directions, while the print bed can move in the z-direction. In general, biomaterials with viscosities between 30 mPa·s and 6 × 10^7^ mPa·s have been demonstrated to be compatible with extrusion bioprinters: lower-viscosity materials provide a more suitable microenvironment to support cell growth, whereas higher-viscosity materials often guarantee more desirable mechanical properties and thus better structural support for the bioprinted models [35]. Moreover, the temperature can be set and controlled during the printing process, which is particularly useful for thermosensitive hydrogels [36]. Cell viability, after the printing process, is lower than those with the previously mentioned bioprinting methods, achieving a survival rate of 40–85%, depending on the extrusion pressure and on the gauge of the nozzle used, but could be even above 90% if the printing parameters are optimized. Proper cell viability, which is fundamental for functional tissue, can be obtained by reducing the pressure and using a large nozzle, though it decreases the resolution and the print speed [37].

However, the widespread employment of extrusion bioprinting is primarily due to the possibility of using high-viscosity bioink and the capability to realize large-scale 3D models with not only a high number of individual cells (>10^8^ cells/mL) but also with preformed cellular aggregates, such as spheroids [38]. Other benefits include the versatility of this technology, allowing the simultaneous printing of different cells and different biomaterials, and its affordability.

An advanced extrusion-based technique is coaxial bioprinting, which consists of the fabrication of complex in vitro tissues through a concentric deposition of biomaterials. In the cancer research field, coaxial bioprinting is particularly interesting when recreating an in vitro vasculature. Through this method, Cao et al. [39] realized a tumor-on-chip model with a hollow blood vessel and a lymphatic vessel pair. Moreover, considering tumor heterogeneity, this technique can be useful for creating multi-compartmental constructs. In this context, Wang et al. [40,41] developed different heterotypic glioma models, including cancer and stromal cell multi-compartmental constructs.

Extrusion bioprinting can be helpful in reproducing gradient structures typical of the tumor microenvironment. In this context, Kuzucu et al. [42] employed a mechanical mixer to combine in a specific way different bioinks in order to form anisotropic tissues. In particular, researchers created a hydrogel characterized by gradients of stiffness, cells, and cell-adhesion peptide concentrations.

**Table 1:** Comparison of different bioprinting methods.

Method	Ink Viscosity	Speed	Resolution	Cell Density	Cell Viability	Cost	Advantages	Disadvantages	Ref.
Stereolithography	<5 Pa·s	fast	25 μm	10^6^–10^7^ cells/mL	>85%	high	no shear stress on cells high accuracy complex structures print speed multimaterial integration	possible cytotoxicity only photocurable biomaterials high cost	[19,20,21,22,23]
Inkjet-based bioprinting	3.5–12 mPa·s	fast	30–50 μm	<10^6^ cells/mL	70–95%	low	high cell viability print speed affordability high throughput	only low viscosity low cell density non-uniform droplet size difficult to print multiple cell types	[24,25,26,27,28,29]
Laser-assisted bioprinting	1–8000 mPa·s	fast	10–100 μm	10^7^–10^8^ cells/mL	>95%	high	no shear stress on cells high cell densities high accuracy high throughput print speed	possible cytotoxicity high cost time-consuming ribbon preparation	[24,30,31,32,33]
Extrusion-based bioprinting	6–30 × 10^7^ mPa·s	slow	200–1000 μm	10^8^ cells/mL	40–85%	medium	high cell densities high-viscosity biomaterials thermosensitive biomaterials photocurable biomaterials multimaterial structures gradient structures vascular/tubular structures	shear stress on cells pressure-induced cell damage lower print speed lower resolution	[34,35,36,37,38,39,40,41,42]

## 3. Bioprinting Requirements and Materials

**Figure 3:**
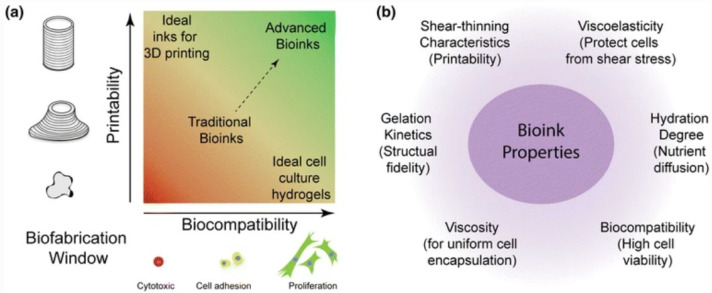
Bioink requirements. (**a**) Schematic representation of the biofabrication window. (**b**) Bioink general properties. Reproduced/adapted with the permission of [43].

Bioprinting employs bioinks, thus printable hydrogels, which encapsulate cells, growth factors, and nutrients. Therefore, from a biological point of view, hydrogels provide an in vivo-like environment resembling the native tissues’ ECM [13]. However, suitable biomaterial should be chosen by considering biological and biomechanical aspects and, eventually, modified according to the tissue-specific features (Figure 3a). The choice of biomaterial is one of the main relevant aspects: it should ensure a high cell viability and proliferation, with an appropriate hydration degree and a correct nutrient supply, in order to biologically achieve a particular TME (Figure 3b) [35,43]. From a structural point of view, bioink printability constitutes a fundamental requirement, as bioprinted models must be able to be printed in order to reproduce the complex microarchitecture of native tissues in vitro in sufficient resolution. It is generally characterized in terms of the controllable formation of extrusive droplets and filaments and their morphology and their shape fidelity after the deposition. Printability, or specifically bioprintability, depends on the rheological properties of the polymeric hydrogel-based bioink and the presence of cells [44]. In this section, the bioprintability requirements for the most popular bioprinting methods and the most used biomaterials will be described. It is important to highlight that the mechanical properties of bioprinted models are dynamic features since they depend on the culture time as well as the extracellular matrix deposition and remodeling by the cancer cells [5].

### 3.1. Bioprintability

Bioinks must fulfill different physicochemical requirements, which affect the printability of the material, the shape fidelity of the model and the cell’s functionality and behavior [45]. In general, printability refers to the capability of forming a 3D structure with acceptable integrity and shape fidelity, and its definition depends on the specific bioprinting method. In extrusion bioprinting, printability is the capability of printing continuous filaments with controllable diameters and defined morphology in a layer-by-layer manner. In order to develop a valid bioink, the simultaneous need for these opposing requirements led to the conceptualization of the biofabrication window, the range of material properties suitable for both printability with high shape fidelity and to support cell viability and function (Figure 3a) [35,43,46]. For the inkjet strategy, printability refers to the ability to generate well-defined droplets in the air. In laser-based printing, a bioink could be defined as printable if a well-defined jet is produced and forms correct droplets. Conversely, in stereolithography, there is no specific definition other than to be able to form a layered structure according to precise instructions.

As previously mentioned, rheological properties greatly impact the bioprintability of a hydrogel precursor (Figure 3b). First of all, the flow behavior of a bioink is related to its resistance to flow, thus, its viscosity. At a given temperature, Newtonian fluids exhibit a constant viscosity with respect to the shear rate, whereas non-Newtonian shear-thinning ones show a viscosity decreasing with increasing shear rate [47]. Bioinks with sufficient viscosity can hold the encapsulated cells in position, preventing inhomogeneity and sedimentation. At the same time, for extrusion bioprinting, a too-large viscosity can hinder the extrudability of the filament. A bioink should also exhibit viscoelastic behavior when undergoing deformation, especially in nozzle-based bioprinting approaches. In non-Newtonian viscoelastic materials, the shear stress needed to make the bioink flow is called yield stress, which must overcome the other involved forces, such as gravity, surface tension and capillarity, to allow the breaking of the elastic network and the deformation of the bioink, forming filaments or droplets [48,49]. Extruded bioink also demonstrated thixotropic behavior; therefore, when constant shear stress is applied, the viscosity decreases with time, yet when this stress is removed, a gradual recovery occurs. This enables the bioink to exhibit low viscosity inside the nozzle tip during extrusion and to maintain a stable shape after the printing process. Viscoelasticity is also important in laser-based bioprinting since a well-defined jet can be formed with the remaining bioink attached to the ribbon [50]. Bioinks employed in inkjet bioprinting should present a low viscosity to easily pass through the ejection system, avoiding nozzle clogging. Moreover, in this technology, the biomaterials should be rheopectic, thus exhibiting a time-dependent behavior, resulting in enhanced viscosity during the shear, which allows droplet formation [50,51]. Differently, in stereolithography, bioinks do not need to exhibit shear-thinning behavior, and a low-viscosity hydrogel can help the removal of unreacted bioink, improving the shape fidelity of the model and avoiding artifacts. In particular, the success of the prints also depends on the curing depth to ensure crosslinking and integration at the interface between two adjacent layers [52].

Other important physical properties of a bioink are surface tension and wettability [53]. The surface tension of a bioink is the internal force exerted on a unit-length border, delimiting the bioink surface, while wettability refers to the capability to maintain contact with solid surfaces, and it is quantified through the contact angle. In particular, for the extrusion-based method, a suitable wettability between the bioink and nozzle facilitates the passage through the nozzle. In addition to this, low adhesion and surface tension allow detachment from the nozzle tip surface, enabling filament deposition [49]. The biomaterials employed for laser bioprinting should exhibit sufficient adhesion and low surface tension characteristics in order to uniformly adhere to the intermediate layer, thus avoiding dripping.

Furthermore, the gelation kinetics also impact the shape fidelity of the bioprinted model, which depends on the crosslinking method. Independently from the bioprinting technique, a rapid in situ gelation is required to retain the shape without spreading [49,54]. However, in droplet-based methods such as inkjet or laser-assisted bioprinting, other considerations, for example on breakup time, must be done. In this context, Yan et al. demonstrated that either the increasing polymer molecular weight or the increasing concentration has a significant effect on the droplet formation process during the inkjet process by increasing the breakup time, decreasing the primary droplet speed and the number of satellite droplets [55]. Moreover, Zhang et al. [56] classified the deposition process during laser bioprinting into three types for the well-defined jetting regime based on the jet/droplet-impingement type: droplet-impingement printing, jet-impingement printing with a single breakup, which offers the best printing quality, and jet-impingement printing with multiple breakups. The distance between the ribbon coating and the receiving substrate also influences the print quality. For example, by lowering this distance, it may be possible to switch from multiple breakup jet-impingement printing to a single breakup one.

However, cells occupying a specific volume in the polymeric solution potentially affect the rheological properties of a bioink and, consequently, also its printability. Numerous studies have been carried out to investigate printability in cell-laden bioinks. For example, Skardal et al. [57] observed a variation in the gelation time in an extruded hyaluronic acid bioink that was comparable to the cell-free counterpart, up to 25 × 10^6^ cells/mL, and longer when up to 100 × 10^6^ cells/mL, while for cell densities, for the majority of 250 × 10^6^ cells/mL, crosslinking did not occur. In regards to the stereolithographic strategy, the effect of the embedded cells on shape fidelity has received only a little attention and additional research is required. However, it is known that bioink viscosity should be viscous enough to prevent cell sedimentation [58]. Furthermore, Xu et al. [59] studied the effects of cell concentrations on the droplet formation process during the inkjet bioprinting of alginate bioinks and observed that, by increasing the cell concentration, the droplet size and velocity decreased, satellite droplets did not form, and the breakup time increased. Zhang et al. [56] found that the addition of living cells in a bioink transforms the printing type of jet-impingement printing to droplet-impingement printing during the laser technique. Non-ideal jetting behaviors have been observed, which might be attributed to the local nonuniformity and no homogeneity of cell-embedded bioinks.

### 3.2. Bioink Materials

Bioinks are based on biomaterials, including those of both natural and synthetic origins. Naturally derived materials are easily obtained from both animal and vegetal resources and generally exhibit good biocompatibility and non-toxicity, offering a better biological environment for cell growth [60]. Natural biomaterials, such as collagen, are a common choice, as they possess the most abundant protein of mammalian cells and can mimic the natural ECM [61]. In addition, it is known that malignant tumors tend to secrete collagen, leading to stiffness and mechanical cues, EMT, and invasion [6]. Other natural biomaterial-based bioinks, such as gelatin, derived from the hydrolysis of collagen; hyaluronic acid (HA) [62,63], another main component of ECM; and Matrigel [64], can closely mimic in vivo conditions. Furthermore, despite several biological advantages, natural polymers do not generally exhibit strong biomechanical properties, which are extremely impactful on cell behaviors; therefore, these biomaterials can be synthetically modified, preserving matrix biocompatibility while allowing greater control over composition and mechanical properties [60]. Gelatin and hyaluronic acid can be modified with methacryloyl groups, forming GelMA and HAMA, respectively, which are photocurable bioink materials whose stiffness can be easily tuned not only through the intensity and the time of light exposure but also through the degree of methacrylation [65]. In this regard, Xue et al. [66] developed a bioink composed of these two biomaterials to obtain a pancreatic cancer model that was able to induce adipose tissue atrophy. Fibrinogen is a large, fibrous, and soluble glycoprotein involved in blood coagulation when it is converted into fibrin by thrombin in the presence of calcium ions [67]. Due to its poor rheological properties, fibrinogen is often combined with other biomaterials, such as alginate and gelatin, to obtain an extrudable bioink, while its low viscosity makes it suitable for inkjet bioprinting. Alternatively, decellularized extracellular matrices (dECMs) offer the advantage of recreating native microenvironments without compromising tissue-specific architecture and the ECM, generating models really close to in vivo conditions, as shown in the study by Yi et al. [68]. In addition to this, it represents one of the few biomaterials printable without being combined with other components [60]. Matrigel is another material belonging to decellularized tissues. Bioinks can be obtained also by vegetal sources. Alginate, derived from brown seaweed, is a non-toxic, biodegradable, and non-immunogenic linear polysaccharide that crosslinks in the presence of calcium and other divalent ions. Its similarity with the glycosaminoglycans found in the native ECM makes this material very attractive in tissue engineering [69,70]. However, being an inert biomaterial, alginate is often functionalized in order to improve cell adhesion and proliferation, as shown in the work of Jia et al. [69], where it was combined with the RGD complex to bioprint an adipose-derived stem cells (ADSCs)-laden matrix, obtaining, in comparison with a simple alginate hydrogel, a more spreading morphology of cells. Agarose, another plant-derived polysaccharide, obtained by red algae, has a structure similar to the ECM due to its macromolecular properties [71]. Thus, it is often used as a bioink component to provide a support structure for cells. Agarose is not as cell-friendly as alginate, yet Forget et al. [72] managed to switch its native form to a carboxylated one, improving not only the mechanical properties but also cell viability after the bioprinting process.

Synthetic polymers are often used with the aim of manufacturing better hydrogels from a mechanical-structural point of view. However, they generally exhibit some limitations, such as the use of toxic solvents and difficulty in encapsulating cells. For this reason, only around 10% of polymers used in bioprinting are synthetic [73]. Among these, only a few can be strictly considered real bioinks, and they are mainly employed as additive biomaterials. For example, polyethylene-glycol (PEG) is characterized by high hydrophilicity, biocompatibility, and low immunogenicity, which make it a suitable material for the scaffold. Nevertheless, like most other synthetic polymers, it does not possess sites for cellular recognition and other biological cues found in natural ECMs for promoting cellular proliferation and differentiation. For this reason, PEG has been coupled with gelatin, GelMA, fibrinogen, and other RGD-containing ECM proteins [74]. Metalloproteinase (MMP), or elastase-sensitive PEG hydrogels, have also been reported. Differently, to enhance printing properties, some acrylated forms of PEG, such as PEGDA, have been employed, which are suitable for most bioprinting technologies. In addition, since the printing temperature with living cells must not exceed 37 °C, some synthetic polymers, such as pluronic acid, cannot be employed as bioink due to their high melting temperature but only as a sacrificial material [73,75]. This last topic is really interesting in cancer research since it could enable the printing of a vascular system, which is extremely important for having more realistic models to investigate metastatic processes.

The main properties of the different bioink materials employed as matrix substrates are summarized in Table 2.

## 4. Bioprinted Diseases Models

The major disadvantage of current cancer models deals with the unsatisfactory imitation of pathophysiological conditions and the associated cell functionality within these 3D tumor models [13]. Biofabrication, through the printing of biomimetic constructs as tumor models with peculiar cellular and ECM compositions, can significantly improve the relevance of such disease models in cancer research. Three-dimensional bioprinting can produce a native tissue-like model by spatially patterning different cell populations to resemble the in vivo microarchitecture. Three-dimensional bioprinted constructs potentially have a physiological microarchitecture and microenvironment, which define the functionality of the in vitro tissue [14,24,31,72]. As will be illustrated in the following section, some bioprinted models of cancer have focused exclusively on the behavior of cancer cells though homocellular constructs; some have taken into account the impact of stromal cells in heterogeneous constructs more similar to native ones, while others aimed to study the cellular epithelial-to-mesenchymal transition and invasive capabilities, and still others managed to incorporate a perfusion component through the use of bioreactors/microfluidic devices or the development of tubular and branching structures, including ECs reproducing the intraluminal blood flow and thus, giving a dynamic and more realistic appearance to these models. Finally, most of such models have been used to carry out drug testing (Table 3).

### 4.1. Breast Cancer Models

Breast cancer is the most diagnosed cancer in women worldwide, with approximately 2.3 million new cases in 2020, according to the WHO [1]. Most cancer-related deaths result from breast cancer metastasis to other organs and tissues, indicating the importance of timely diagnosis and efficient treatment. Therefore, reliable breast cancer models are increasingly needed.

Campbell et al. [76] used a modified thermal inkjet bioprinter to print the breast cancer cell line, MCF-7, within sodium alginate. After the bioprinting process, an alteration in different genes was observed, hallmarks of more aggressive properties that are implicated in cancer metastasis, demonstrating that bioprinted cells can potentially improve the in vitro models for drug discovery. Consequently, other tumor cell lines, such as MDA-MB-231 and healthy breast cells, MCF-10A, were thermally bioprinted and exposed to a combination of palbociclib and letrozole with or without radiotherapy treatment [77]. Researchers found that bioprinted cells showed radioresistance similar to the 2D cultures but a higher resistance to the combo treatment, especially in the MCF-7 cell line.

It is known that the ECM composition plays a fundamental role in the cells’ behavior, and in this regard, Swaminathan et al. [78] developed bioprinted models, encapsulating both the preformed spheroids of human breast epithelial cell lines (healthy and tumorigenic) and individual cells in three different bioinks: Matrigel, gelatine-alginate, and collagen-alginate. Individual breast cells spontaneously formed spheroids in the Matrigel-based bioink, suggesting that the major Matrigel protein compound, laminin, represents a crucial element in breast epithelial spheroid formation. Preformed breast spheroids maintained their structure, polarization, viability, and function after bioprinting and remained more resistant to chemotherapeutic agents, such as paclitaxel, than the individual cell-based bioprinted constructs.

To mimic the adipose component of the breast tissue, Chaji et al. [79] bioprinted multicellular cell-laden hydrogels composed of breast cancer cells and adipocytes, using an optimal combination of alginate and gelatin, and managed to mimic the tissue stiffness observed in a physiological breast cancer tumor environment. The results showed that cancer cells in the co-culture with adipose cells tended to form clusters within the central zone of the 3D model. In this area, researchers found both viable and dead cells, suggesting necrotic behavior, which is typical of the hypoxic microenvironment.

Recent studies have demonstrated that breast tissue-derived matrices could be an important biomaterial for recreating the complexity of the tumor microenvironment. For instance, the breast cancer organoid models developed by Mollica et al. [80], composed of breast cancer cells and human mammary tissue dECM, recapitulated biologically relevant features, providing a platform to study cancer initiation and its progression and for investigating the response of tumor cells to different treatments. Differently, Blanco-Fernandez et al. [81] engineered a decellularized porcine breast tissue with alginate and GelMA to obtain a printable and cytocompatible bioink. The novel biomaterial was used to print breast cancer models with MCF-7 cells in the inner core, surrounded by stromal cells. The tumor cells exhibited high cell viability, proliferation, tendency to aggregate into spheroids and demonstrated drug resistance when compared to the 2D cultures.

**Figure 4:**
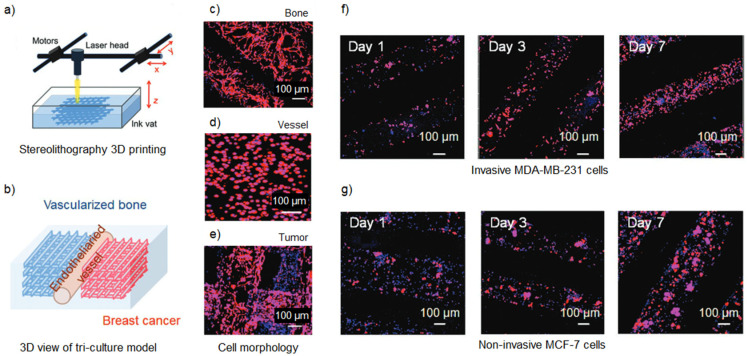
Vascularized breast-to-bone metastatic cancer model. (**a**) Laser-based stereolithographic 3D bioprinting scheme (**b**) Tri-culture model scheme, with breast cancer-laden region, endothelialized vessel, and bone region. (**c**) Morphology of hFobs in the bone region. (**d**) Morphology of ECs in the vessel region. (**e**) Morphology of MDA-MB-231 cells in the tumor region. (**f**) Morphology of MDA-MB-231 cells at days 1, 3, and 7. (**g**) Morphology of MCF-7 cells at days 1, 3 and 7. Reproduced/adapted with the permission of [84].

Several researchers have developed models to study the phenomenon of bone metastasis since the bone is the most common site of colonization for breast cancer cells. A biomimetic bone matrix GelMA-based model was developed by Zhou et al. [82] with a stereolithographic bioprinter to investigate the effects of breast cancer on bone stromal cells. The results demonstrated that co-culturing the breast cancer cells (MDA-MB-231) with mesenchymal stem cells (MSCs) or osteoblasts in bioprinted models better stimulated the secretion of VEGF compared to a monoculture of tumor cells. Additionally, the growth of breast cancer cells was improved during the co-culture. Exploring the crosstalk between different cell types, Zhu et al. [83] carried out a study observing the interaction between human fetal osteoblasts (hFOBs) and metastatic breast cancer cells on a PEGDA-bioprinted artificial bone matrix, functionalized with nano-hydroxyapatite (nHA). The tumor cells co-cultured with bone cells impacted the morphology and proliferation rate of both cell types and enhanced IL-8 secretion, an angiogenic and tumorigenic factor. Moreover, the cancer cells’ proliferation rate was observed to be proportional to the concentration of nHA. Such results suggest that the composition of the 3D-bioprinted matrix is fundamental to reproducing the in vivo behavior of metastatic cancer cells in the bone microenvironment. In the research work carried out by Cui et al. [84], a vascularized breast-bone metastatic model was bioprinted using stereolithography with optimized GelMA-PEGDA inks (Figure 4a). In particular, this proof-of-concept model was made from an endothelialized vessel interposed between two grid structures: one for the breast cancer matrix and the other for the bone-like matrix, mineralized by nano-hydroxyapatite (Figure 4b), which allowed an appropriate morphological development (Figure 4c–e). The results showed a promising capacity of this model for studying breast cancer invasion in bone (Figure 4f,g).

**Figure 5:**
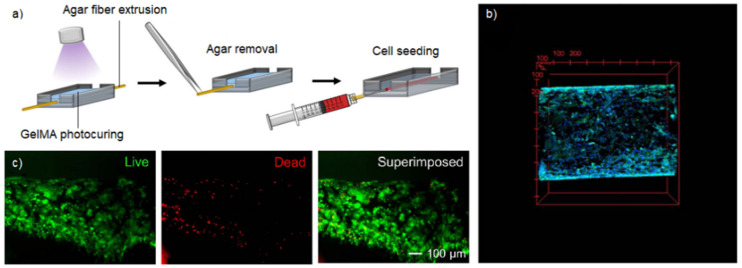
Ductal carcinoma model. (**a**) The main steps of sacrificial bioprinting: an agarose microfiber was extruded onto a GelMA layer previously crosslinked and covered with another GelMA layer, followed by the photo-crosslinking of the entire construct and the removal of the agarose microfiber to induce the formation of a duct-like structure; finally, cells were seeded into the microchannel. (**b**) Confocal microscopy 3D image of the ductal carcinoma model after 14 days of culture. Nuclei staining in blue. (**c**) Dead/alive staining of MCF-7 cells after 24 days of culture. Reproduced/adapted with the permission of [85].

To resemble the entire mammary duct structure, a proof-of-concept ductal carcinoma model was realized by Duchamp et al. [85] with the sacrificial bioprinting technique (Figure 4a). Breast cancer cells, seeded into a GelMA microchannel, exhibited various features typical of ductal carcinoma, such as proliferation, metastatic potential, a heterotypic structure, and deposition of basement membrane molecules (Figure 5b,c).

### 4.2. Lung Cancer Models

Lung cancer is the leading cause of cancer death worldwide [1]. Non-small cell lung cancer, with a high metastasis rate and high recurrence rate, accounts for about 80–85% of lung cancers and still lacks effective clinical treatments [86]. For this reason, it is necessary to create reliable and more realistic tumor models to study cancer behavior and develop and test new therapies.

For the most highly aggressive tumors, such as non-small cell lung cancer, one of the biggest challenges is to deeply understand the invasion and metastasis mechanisms. In this regard, a bioprinted lung cancer model with A549 and 95-D cells was successfully developed by Wang et al. [87]. The bioink—realized with alginate and gelatin—allowed for high cell viability up to 12 days of culture and structural integrity of the entire construct. Through the expression of the metalloproteinases MMP-2 and MMP-9 genes and the scratch test, researchers concluded that the cells in the three-dimensional model showed higher invasion and migration capabilities compared to the traditional 2D culture model.

To understand the influence of the stromal component, Mondal et al. [88] optimized the rheological parameters of an alginate-gelatin bioink to easily print non-small cell lung tumor cells and lung cancer-associated fibroblasts. This bioprinted construct guaranteed high cell viability and allowed the formation of spheroids and the promotion of the epithelial–mesenchymal transition with the downregulation in E-cadherin and the upregulation of vimentin and α-SMA.

These last two works developed a bioink based on a higher gelatin component than the alginate one, while Yang et al. [89] developed a bioink based mainly on alginate to be used with A549 cells. The lung cancer model—produced through extrusion—was employed for the drug testing of eight different traditional Chinese medicines, showing a higher drug resistance compared to the monolayer cell culture for most of them. This result provided a new strategy to screen antitumoral drugs at the tissue level, an alternative to animal testing.

### 4.3. Brain Cancer Models

Glioblastoma represents a type of brain tumor characterized by high resistance to different chemotherapeutic agents. In addition to the overall poor effect of chemotherapy on glioblastoma, intratumoral heterogeneity negatively impacts patient survival. In this context, bioprinted brain tumor models for drug screening and personalized medicine can be particularly beneficial [1].

The first bioprinted model of glioblastoma was realized by Dai et al. [90] using a gelatin/alginate/fibrinogen hydrogel to encapsulate glioma stem cells. Glioma stem cells demonstrated both characteristics of stemness and differentiation potential, with a higher expression of VEGF. Moreover, this 3D-printed tumor model showed more resistance to temozolomide than the 2D cultures, better mimicking the in vivo cancer response.

**Figure 6:**
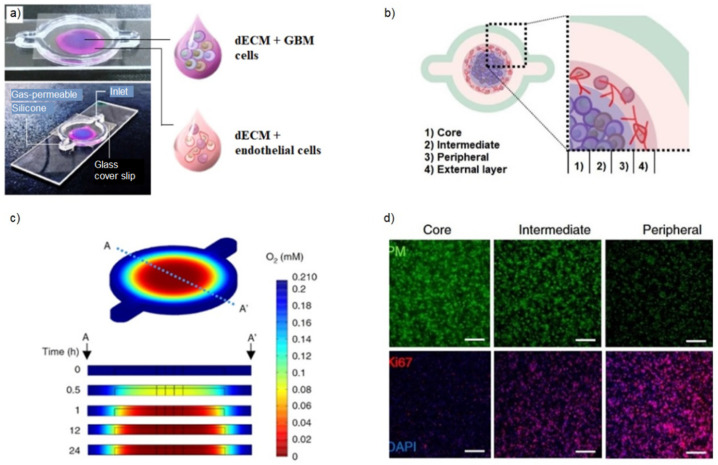
Glioblastoma-on-a-chip model. (**a**) Photographs of the GBM-on-a-chip model, including the dECM bioink laden with GBM cells (blue) or HUVECs (magenta), printed within a gas-permeable silicone on a glass slide and covered by a glass slip. (**b**) Compartmental subdivision of the bioprinted model in core, intermediate, and peripheral areas with GBM cells and external layer with HUVECs. (**c**) Oxygen level simulation within the bioprinted hydrogel, carried out with COMSOL Multiphysics. (**d**) Immunostaining images of core, intermediate, and peripheral zones employing pimonidazole (PM) for the hypoxic cells, Ki67 for the oxygenated proliferating cells, and DAPI for the cell nuclei. Scale bar, 200 μm. Reproduced/adapted with the permission of [68].

However, the stromal component strictly impacts glioblastoma, providing mechanical and biological cues for tumor progression through crosstalk with tumor cells. To investigate the cellular interactions between the glioblastoma cells and glioblastoma-associated macrophages, Heinrich et al. [91] developed a 3D-bioprinted GelMA/gelatin-based mini-brain, employing a two-step bioprinting method. In this model, glioblastoma cells recruited the macrophages, which, in turn, induced tumor cell progression and invasiveness. The bioprinted mini-brain was also used as a tool to test specific chemotherapeutic drugs for glioblastoma, such as carmustine and immunotherapies. Yi et al. [68] fabricated a bioprinted glioblastoma-on-a-chip, recreating a cancer-stroma concentric-ring structure (Figure 6). For this purpose, two different extrudable bioinks were formulated, separately mixing a porcine-decellularized extracellular matrix with patient-derived tumor cells and vascular ECs (Figure 6a). This compartmentalized model was able to sustain a radial oxygen gradient, mimicking the structural, biochemical, and biophysical characteristics of the native tumor microenvironment (Figure 6b–d).

Tang et al. [92] also developed a compartmentalized in vitro glioblastoma using GelMA and glycidyl methacrylate-HA (GMHA) hydrogels, where the peripheral part was composed of astrocytes, which comprises approximately half of all brain cells, and the inner core incorporated the brain cancer stem cells. This model was produced through a stereolithographic bioprinter with a digital micromirror device chip that enabled the photo-crosslinking of the two cell-embedded biomaterials, modulating the stiffness of the tumor core in such a way as to have a higher modulus than the outer healthy region. A recent study conducted by Hermida et al. [93] aimed to resemble the heterogeneous glioblastoma tumor microenvironment in bioprinted RGD-alginate/collagen/HA models using a multi-nozzle extrusion bioprinter, including cancer and stromal cells, thus providing a more realistic chemotherapeutic response compared to the 2D monolayer.

A main feature of glioblastoma is the large-scale abnormal vascularization involved in the rapid spread of the tumor. In this regard, Wang et al. [40] employed the coaxial bioprinting of alginate to produce shell–core hydrogel microfibers containing glioma stem cells (GSC23) in the outer part and glioma cells (U118) in the inner one, exhibiting good cell viability and proliferation. Additionally, in the core, an increase in the expression of endothelial growth factor receptor-2 (VEGFR2), matrix metalloproteinases, and other factors related to invasion and drug resistance were observed, concluding that this coaxially bioprinted model could be a reliable platform to mimic the glioblastoma microenvironment. The same bioprinting approach was adopted by other further studies, fabricating a shell-glioma cell (U118)-/core-human umbilical vein endothelial cell (HUVEC) model, where it was possible to observe that U118 cells promoted the angiogenesis by secreting vascular growth factors [41]. The same research group showed that glioma stem cells in a novel bioprinted gelatin/alginate model exhibited a high capability to form tumor spheroids, secrete vascular growth factors, and form vessel-like structures in vitro [94].

In an interesting study, Neufeld et al. [75] recapitulated the tumor microenvironment by engineering fibrin glioblastoma bioink comprising patient-derived glioblastoma cells, astrocytes, and microglia. In addition, a perfusable vasculature was developed by employing a sacrificial pluronic bioink coated with ECs and pericytes. Glioblastoma cells, maintained in culture for 8 weeks after printing, showed high proliferation and migration capabilities, validating this 3D-bioprinted model as a promising tool for rapid and reliable preclinical studies.

### 4.4. Ovarian and Cervical Cancer Models

Gynaecological cancers are female tumors that mainly affect the ovaries and the uterus. Ovarian cancer comprises five different histological subtypes, and in particular, high-grade serous carcinoma is the most commonly diagnosed ovarian cancer, showing a high drug resistance to platinum-based chemotherapy. Moreover, it is typically diagnosed at a late stage and still has no effective screening strategy. Cervical cancer is the fourth most frequently diagnosed cancer and the fourth leading cause of cancer death in women, with an estimated 604,000 new cases worldwide in 2020 [1]. For this reason, there is an urgent need to develop reliable in vitro models.

However, bioprinting technology is still poorly investigated in the context of bioengineering tumors of reproductive tissues. Xu et al. [95] developed a three-dimensional co-culture model of ovarian cancer cells (OVCAR-5) and normal fibroblasts (MRC-5) encapsulated in microdroplet-based Matrigel bioink. The viability of the ovarian cancer cells was not affected by the printing process inducing the spontaneous formation of tumoroids, which could provide a platform for efficient ovarian tumor drug testing. Recently, Baka et al. [96] established a high-throughput model of ovarian cancer, bioprinting cancer cells (SKOV-3) and cancer-associated fibroblasts embedded in an optimized gelatin-alginate hydrogel. The results demonstrated that cells in the structure’s periphery were more proliferative compared to the core since they are more exposed to nutrients and oxygen, suggesting the establishment of a gas and nutrient gradient over the bioprinted structures that represents one of the main features of the native tumors. Moreover, Wu et al. [97] designed an artificial ovary using a GelMA bioink, demonstrating the suitability of the hydrogel for the growth and maturation of different ovarian tumor cell lines.

Zhao et al. [98] fabricated a cervical tumor model employing a gelatin/alginate/fibrinogen bioink containing Hela cells. First, it was demonstrated that cervical cancer cells formed round spheroids within the 3D hydrogel, differently from the 2D culture, where they showed a flat and elongated morphology. Then, tumor cells in the 3D-bioprinted model displayed a higher MMP expression and a higher chemoresistance to paclitaxel than the 2D cultures, better mimicking the in vivo tumor microenvironment. A similar study was recently carried out by Gospodinova et al. [99] by formulating a novel bioink composed of hydroxyethylcellulose (HEC) and alginate. Differently from Zhao et al., the paclitaxel treatment resulted more efficiently than the control, indicating that bioprinted models exposed to paclitaxel provided a favorable environment for drug diffusion. Moreover, the drug’s cytotoxic effect was more pronounced on the cells embedded in the hydrogel than those in the 2D culture. The same cell line was employed by Pang et al. [100] to investigate the epithelial–mesenchymal transition in an advanced bioprinted cervical tumor model through the addition of TGF-β. The tumor cells embedded in an alginate/gelatin/Matrigel-based hydrogel showed rapid proliferation, the formation of spheroids, the downregulation of epithelial markers such as E-cadherin, and the upregulation of several mesenchymal ones.

### 4.5. Liver Cancer Models

Liver cancer is the sixth most commonly diagnosed cancer and the third leading cause of cancer death worldwide in 2020 [1]. Today, models that accurately represent inter-patient variation and heterogeneity of the human liver tumor represent a great challenge. However, some different 3D-bioprinted models were developed to study the behavior of this tumor and the reaction to different therapies.

Xie et al. [101] successfully established a liver tumor model, bioprinting patient-derived hepatocellular carcinoma cells in gelatin/alginate ink, which can resemble several features of the native tumor. The tumor cells stably maintained the typical features of the original tumors during long-term culturing. In addition to this, the tumorigenic potential of hepatocarcinoma cells transplanted into a BALB-nude mouse was retained after long-term culturing. Finally, this in vitro construct demonstrated its suitability as a personalized model for several anticancer drug screenings.

Zhou et al. [102] fabricated a model consisting of the hepatocarcinoma cell line, HepG2, and alginate/gelatin/fibrinogen hydrogel, through a custom-made bioprinter to test different anticancer drugs. In particular, they investigated the effects of 5-fluorouracil (5-FU), mitomycin (MMC) and their combination. Researchers found that the HepG2 resistance to mitomycin was weaker; however, their resistance to 5-fluorouracil was stronger compared with that in the 2D culture. Combining the two drugs, they concluded that MMC had the main effect during the first 24 h, while the 5-FU produced effects later.

**Figure 7:**
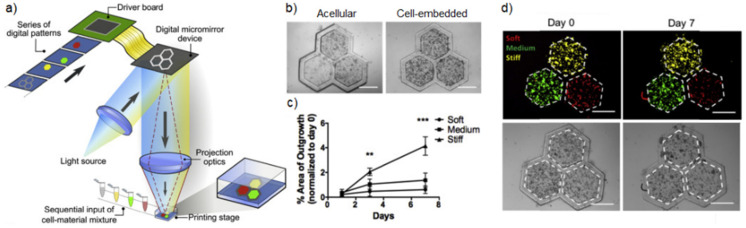
Heterogeneous stiffness liver cancer model. (**a**) Schematic representation of digital light processing-based bioprinting employed to fabricate the liver cancer model. (**b**) Bright-field images showing bioprinted cell-free and cell-laden hydrogels. Scale bar: 500 μm. (**c**) Plot showing the percent area of cell invasion deriving from the three different hydrogels over 7 days; ** *p* ≤ 0.01, *** *p* ≤ 0.001. (**d**) Merged fluorescence (**top**) and bright-field (**bottom**) images showing the HepG2 cell locations relative to their regions at day 0 and day 7; red, green, and yellow represent the soft, medium, and stiff matrices, respectively. Scale bar, 500 μm. Reproduced/adapted with the permission of [103].

To investigate the impact of stiffness on cancer cells’ behavior, Ma et al. [103] bioprinted a photocurable porcine-decellularized liver tissue through a light-based process, tuning the mechanical properties depending on the region of the model in order to have a biomimetic, heterogeneous modular structure. When cultured in a hydrogel with diseased stiffness, the HepG2 cells demonstrated a lower growth but increased invasiveness compared to the healthy controls (Figure 7c). Furthermore, this bioprinted construct, characterized by varied stiffness, managed to obtain the cancer cells’ invasion from the nodule with the highest stiffness (Figure 7d).

Mao et al. [104] embedded human intrahepatic cholangiocarcinoma (ICC) cells into a composite hydrogel system of gelatin/alginate/Matrigel to bioprint a biomimetic tumor microenvironment. The cancer cells within the three-dimensional model had a high survival rate and proliferation and presented a high level of matrix metalloproteinase protein, inducing metastatic behavior. Finally, the anticancer drug resistance of the tumor cells in the 3D-bioprinted platform proved their stem-like properties and revealed a tool with good potential to study tumorigenesis and the development of new target therapies.

The tumor microenvironment significantly affects tumor progression, metastasis, and drug response. In this perspective, Li et al. [105] constructed a bioprinted model characterized by a central zone containing cholangiocarcinoma cells and a peripheral one with tumor-associated stromal cells, including macrophages, fibroblasts, and endothelial cells. Researchers found a higher proliferation in the co-culture compared to both the 2D culture and 3D monoculture. Moreover, the heterotypic model had a greater ability of invasion, and its drug resistance demonstrated the stemness-like properties of cancer cells.

As well as the tumor microenvironment components’ impact on cancer cells, interstitial fluid flow, mimicked by a perfused culture, affects their behavior, achieving a more realistic in vivo biological environment. In this regard, a proof-of-concept dynamic in vitro liver cancer model was recently developed by Moss et al. [106], with a custom-made bioprinting system incorporating cancer cells, endothelial cells, other hepatic stromal components, such as stellate (hSCs) and Kupffer cells (KCs), and human adipose microvessel fragments (haMVs) in a collagen-based bioink, which showed to improve the liver model’s outcomes as compared to an absence of haMVs. Furthermore, the presence of the haMVs seemed to modify the hepatocyte metabolism in a dynamic culture. Moreover, Li et al. [107] combined the 3D bioprinting technology with microfluidics to study the effect of metuzumab on hepatocarcinoma cells. Hydroxypropyl chitin (HPCH), a thermosensitive hydrogel, and Matrigel were used for bioprinting cancer cells and maintaining the spheroids during the dynamic culture inside the microfluidic device. The results showed an increasing migration and invasion ability and higher resistance to various concentrations of the monoclonal antibody drug compared to both the 2D- and 3D-static printing models.

### 4.6. Pancreas Cancer Models

Pancreatic cancer has the highest mortality rate of all major cancers. It has a poor prognosis, and in fact, the majority of patients diagnosed at a distance have a 5-year survival lower than 10% [108]. Pancreatic cancer is constituted by an extensive and heterogeneous microenvironment, which seriously affects antitumor drug treatment effectiveness. Thus, there is an urgent need for a new reliable in vitro platform to study and possibly find an effective treatment.

Hakobyan et al. [109] developed 3D pancreatic cell spheroids in a GelMA hydrogel, employing laser-assisted bioprinting that described their phenotypic modification over time. The bioprinted model comprising both acinar and ductal cells turned out to be a promising 3D model for the study of the tumorigenesis of pancreatic ductal adenocarcinoma development.

Cancer-associated fibroblasts are involved in tumor growth. In this context, Hou et al. [110] established tumor models using two types of cancer-associated fibroblasts and two different pancreatic cancer cell lines derived from human tumor tissue. Through a 3D magnetic bioprinting technology, they managed to produce organoids without encapsulating the cells in a hydrogel. Such tissue-like constructs were subsequently tested with numerous anticancer-approved drugs.

Cancer-associated cachexia is a multifactorial metabolic syndrome that occurs in patients with different tumors, including pancreas cancer, and during advanced stages, manifesting with inflammation, adipose lipolysis, and weight loss [111]. To understand the fat loss-associated mechanism, Xue et al. engineered and then vascularized a white adipose bioprinted model, based on the GelMA and HAMA bioinks, and cultured it in a pancreatic cancer cell-conditioned medium, showing a remodeling of adipose tissue and enhanced angiogenesis [66].

### 4.7. Colorectal Cancer Models

The WHO describes colorectal cancer as the third most diagnosed tumor worldwide, counting for more than 1.9 million new cases in 2020, and the second cause of cancer death after lung cancer [1]. Unfortunately, colorectal cancer has a poor prognosis, and the 5-year survival rate of colorectal cancer patients is still too low. In this scenario, bioprinting results in a helpful instrument to build reliable in vitro models for biology studies and drug testing purposes.

Sbirkov et al. [112] developed a colorectal cancer model using a commercial RGD-alginate bioink as a platform to test three traditional chemotherapeutics: 5-fluorouracil, irinotecan, and oxaliplatin. Researchers observed that, except for oxaliplatin, the other drugs are less effective in the 3D-bioprinted models than in 2D cultures.

Chen et al. [113] employed surface acoustic waves (SAW)-3D printing to reproduce and study the metastatic mechanism in a colorectal cancer model based on a GelMA bioink containing patient-derived tumor and stromal cells. The drug resistance was evaluated on the clinical drug 5-fluorouracil. The invasion and migration of cancer cells were assessed using the immunofluorescence technique, pre- and post-drug administration, confirming a lower proliferation capability and a high drug resistance after the treatment.

In order to create a more complex colorectal cancer model, Burkholder-Wenger et al. [114] defined two distinct regions with trenches to simulate the longitudinal section of blood vessels, and intestinal villi-like structures, employing an alginate–GelMA-based ink added with cellulose nanocrystals. The same biomaterial was loaded with endothelial (EA-hy-926) and tumor (HCT-116) cells and deposited over the printed acellular structure, showing good adhesion and high viability.

**Figure 8:**
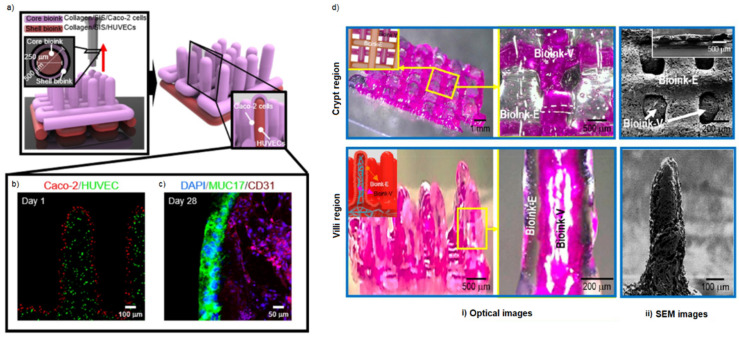
Intestinal model with finger-like villus structure. (**a**) 3D coaxial bioprinting process for producing a core–shell intestinal model with epithelial Caco-2 cells and endothelial HUVECs using collagen/SIS bioinks. (**b**) Cell tracker image of the epithelium region (red) and capillary region (green) on day 1. (**c**) DAPI (blue)/MUC17 (green)/CD31 (magenta) image of the epithelium and capillary regions on day 28. (**d**) Optical (i) and (ii) SEM images of the villi and crypt regions for the bioprinted model. Reproduced/adapted with the permission of [115,116].

Kim et al. [115,116] developed a tumoral intestinal model comprising an epithelium layer and a vascular network, employing two collagen bioinks with different concentrations of tannic acid, containing Caco-2 cells and HUVECs, respectively (Figure 8a). In particular, they used a coaxial nozzle to provide a unique three-dimensional structure, which was characterized by the capillary network surrounded by the Caco-2 cells (Figure 8b,c). Combining the traditional bioink with a percentage of porcine-decellularized small intestine submucosa (SIS), it was possible to obtain a really advanced in vitro model of a pathological intestine with a higher structure resolution (Figure 8d).

**Table 3:** Bioprinted disease models.

Tumor Type	Study Aim	Cell Lines/Types	Biomaterials	Method	Ref.
Breast	gene alterations, drug resistance after radiation therapy	MCF-7, MDA-MB-231	alginate	inkjet	[76,77]
	drug resistance	MCF10A-NeuN, MDA-MB-231, MCF-7	Matrigel, gelatin–alginate, collagen–alginate	coaxial extrusion	[78]
	cancer cells–adipocytes crosstalk	MCF-7, ADSCs	gelatin–alginate	extrusion	[79]
	large organoids/tumoroids production	MCF-7, MDA-MB-468	human dECM + rat dECM	extrusion	[80]
	tissue-derived bioinks tuning, drug resistance	MCF-7, hAMSCs	porcine dECM–alginate–GelMA	extrusion	[81]
	bone metastasis	MDA-MB-231, MSCs/hFOBs	GelMA	stereolithography	[82]
	bone metastasis	MDA-MB-231, hFOBs	PEGDA-nHA	stereolithography	[83]
	bone metastasis	MDA-MB-231, hFOBs, HUVECs	GelMA-PEGDA	stereolithography	[84]
	cell invasion	MCF-7	GelMA + agarose	sacrificial extrusion	[85]
Lung	cell invasion and migration	A549, 95-D	Alginate–gelatin	extrusion	[87,88,89]
	cancer cell–CAFs crosstalk	EGFR T790M, AA0022			
	drug resistance	A549			
Brain	drug resistance	SU3	alginate–gelatin–fibrinogen	extrusion	[90]
	cancer cells–CAFs crosstalk, drug resistance	GL261, RAW264.7	GelMA–gelatin	extrusion	[91]
	hypoxia, drug resistance	U-87, HUVECs	porcine dECM	extrusion	[68]
	cancer cells–stroma crosstalk, drug resistance	CW468, GSC23, GSC3264, GSC2907, M2, hNP1s, astrocytes	GelMA-GMHA	stereolithography	[92]
	drug resistance	U87-MG, WI-38, MM6	RGD-alginate–collagen–HA	extrusion	[93]
	invasion, drug resistance, angiogenesis	U118, GSC23; U118, HUVECs	alginate	coaxial extrusion	[40,41]
	angiogenesis	U118, GSC23	gelatin–alginate	coaxial extrusion	[94]
	vascularization, drug resistance	U-87MG, T98G, U373, HUVECs, MDA-MB-231	fibrin + pluronic	sacrificial extrusion	[75]
Ovary-cervix	co-culture model production	OVCAR-5, MRC-5	Matrigel	inkjet	[95]
	co-culture model production	SKOV, ATCC HTB-65TM	gelatin–alginate	extrusion	[96]
	healthy/cancerous artificial ovaries production	COV434, KGN, ID8, mice ovarian somatic cells	GelMA	extrusion	[97]
	metastatic potential, drug resistance	Hela	gelatin–alginate–fibrinogen	extrusion	[98]
	drug resistance	Hela	HEC-alginate	extrusion	[99]
	Epithelial–mesenchymal transition	Hela	alginate–gelatin–Matrigel	extrusion	[100]
Liver	personalized model production, drug resistance	primary cells	gelatin–alginate	extrusion	[101]
	drug resistance	HepG2	alginate–gelatin–fibrinogen	extrusion	[102]
	stiffness impact on cancer cells’ behavior	HepG2	porcine dECM	stereolithography	[103]
	drug resistance	primary cells	gelatin–alginate–Matrigel	extrusion	[104]
	cancer cells–stroma crosstalk, drug resistance	RBE, HUVEC, CCC-HPF-1, THP-1	GelMA	extrusion	[105]
	microvascularization, drug resistance	rat hepatocytes, ECs, KCs, and hSCs, haMVs	collagen	extrusion	[106]
	co-culture microfluidic model production, drug resistance	SMMC-7721, PUMC-HUVEC-T1	HPCH-GelMa	extrusion	[107]
Pancreas	heterotypic spheroids production	AR42J-B-13	GelMA	laser	[109]
	fat loss due to cachexia	HT-29, PANC-1	GelMA-HAMA	stereolithography	[66]
Colorectum	drug resistance	Caco	RGD-alginate	extrusion	[112]
	cell invasion, migration, drug resistance	primary cells, CAFs	GelMA	inkjet (SAW)	[113]
	villi and trenches model production	HCT-116, EA-hy 926	GelMA–alginate–cellulose	extrusion	[114]
	core–shell villi model production	Caco-2, HUVECs	collagen	coaxial extrusion	[115]
	core–shell villi model production	Caco-2, HUVECs	collagen–porcine dECM	coaxial extrusion	[116]

## 5. Conclusions and Future Directions

The 3D in vitro cancer models better recapitulate the characteristics of real tumors than 2D models, such as cell–cell and cell–ECM interactions, showing greater cell viability and higher chemoresistance to anticancer drugs. In the field of three-dimensional models, bioprinting has the potential for better in vitro modeling of the tumor microenvironment, providing high control over the spatial distribution of cells. High-throughput fabrication helps to better characterize the tumor’s development and response to anticancer therapies. Cancer research studies conducted using animal models exhibit biological differences from humans, resulting in poor clinical outcomes, thus limiting our understanding of cancer behavior in humans. The current bioprinting applications for cancer research represent promising tools for establishing new 3D cancer models to achieve new discoveries in cancer biology or to test clinical drugs. These models could bridge the gap between traditional 2D cultures and xenograft models, recapitulating the spatial complexity of the native tumors and obtaining more reliable clinical outcomes. In particular, in this review, the key aspects that style the fabrication of tumor models as advantageous through 3D bioprinting were highlighted. The printing method was chosen according to a number of requirements, such as the biomaterials used, the desired shape, size, and resolution of the fabricated in vitro models, etc. Bioprinting allows the patterning of different bioinks in a user-controlled manner to form complex 3D constructs with biomimetic tumor microarchitecture. Multiple bioinks with different cell types, both tumor and stromal, can be used for printing functional tumor constructs. Although synthetic hydrogels allow the achievement of higher mechanical properties, the most used bioinks are those of natural origin, given their good or even excellent biocompatibility. However, natural biomaterials, due to their low rheological properties, are rarely used individually, especially in the extrusion process; however, they are often combined with each other to improve printability and shape fidelity. In particular, the most commonly employed bioinks are those made up of alginate and gelatin. GelMA is also widely used, despite its printing requiring particular attention. Moreover, some bioprinting techniques allow the integration of perfusable vasculature within 3D cancer models, better inducing biological and mechanical cues similar to native tumors. In this regard, the production of disease models with specific cellular and ECM compositions can significantly increase the relevance of such bioprinted constructs being employed in the investigation of molecular and biological mechanisms of cancer.

However, there are other aspects related to the tumor microenvironment, still little studied, on which bioprinting could be investigated. For example, recent studies have demonstrated the recruitment of peripheral nerves to the tumor microenvironment. This process, termed tumor innervation, is associated with an aggressive tumor phenotype, metastasis, and poor clinical outcome [117,118]. Moreover, in order to have a real dynamic tumor model, in addition to the inclusion of perfusion elements, 3D bioprinting techniques could move towards the fourth dimension, exploring and using smart polymers that are able to respond to external stimuli, thus mimicking the different stages of cancer by programming the swelling and/or degradation of the hydrogel matrix [119,120].

In conclusion, these rapid and, generally, low-cost biofabrication methods enable us to obtain models adaptable for clinics as diagnostic tools for rapid testing and personalized medicine. Nevertheless, it is necessary to improve the current bioprinting techniques, develop innovative printing materials, and achieve the correct trade-off between the biological and mechanical aspects, in order to produce more complex and realistic in vitro cancer models. 

## Figures and Tables

**Table 2:** Comparison of different biomaterials employed in cancer modeling.

Bioink Material	Type	Advantages	Disadvantages	Ref.
collagen	natural	low-antigenicity biodegradability bioactivity (RGD)	mechanical instability high cost	[60,61]
gelatin	natural	non-antigenicity biodegradability bioactivity (RGD) gelation at low temperatures thermo-reversibility ease of processing low cost	low mechanical properties rapid degradation	[60]
HA	natural	biodegradability bioresorbability hydratation angiogenesis promotion	no cell recognition sites mechanical instability rapid degradation	[62,63]
GelMA	semi-synthetic	biocompatibility tunable mechanical properties	necessity of a temperature-controlled system limited cell activity with high concentrations difficult to print	[65,66]
HAMA	semi-synthetic	biocompatibility tunable mechanical properties	no cell recognition sites	[66]
dECM	natural	biological and mechanical similarities to native ECM printability on its own	difficult preparation high cost availability dependent on human donor variability	[60,68]
Matrigel	natural	biological and mechanical similarities to native ECM	high cost variability limited suitability to clinical translation complex rheological behavior low mechanical properties necessity of a temperature-controlled system	[60,64]
fibrinogen	natural	biodegradability non-immunogenicity bioactivity (RGD) angiogenesis promotion	low rheological properties irreversible crosslinking (fibrin)	[67]
alginate	natural	non-toxicity biodegradability non-immunogenicity inertia low cost	no cell recognition sites	[69,70]
agarose	natural	thermo-reversibility inertia structural similarities with native ECM tendency to gellify	no cell recognition sites	[71]
PEG and derived	synthetic	high hydrophilicity biocompatibility low immunogenicity tunable mechanical properties	no cell recognition sites	[73]

## Data Availability

Not applicable.
